# Quantitative NMR-Based Biomedical Metabolomics: Current Status and Applications

**DOI:** 10.3390/molecules25215128

**Published:** 2020-11-04

**Authors:** Alexandra A. Crook, Robert Powers

**Affiliations:** 1 Department of Chemistry, University of Nebraska-Lincoln, Lincoln, NE 68588-0304, USA; acrook2@unl.edu; 2 Nebraska Center for Integrated Biomolecular Communication, University of Nebraska-Lincoln, Lincoln, NE 68588-0304, USA

**Keywords:** quantitative NMR, qNMR, metabolomics, multidimensional NMR, biomarkers

## Abstract

Nuclear Magnetic Resonance (NMR) spectroscopy is a quantitative analytical tool commonly utilized for metabolomics analysis. Quantitative NMR (qNMR) is a field of NMR spectroscopy dedicated to the measurement of analytes through signal intensity and its linear relationship with analyte concentration. Metabolomics-based NMR exploits this quantitative relationship to identify and measure biomarkers within complex biological samples such as serum, plasma, and urine. In this review of quantitative NMR-based metabolomics, the advancements and limitations of current techniques for metabolite quantification will be evaluated as well as the applications of qNMR in biomedical metabolomics. While qNMR is limited by sensitivity and dynamic range, the simple method development, minimal sample derivatization, and the simultaneous qualitative and quantitative information provide a unique landscape for biomedical metabolomics, which is not available to other techniques. Furthermore, the non-destructive nature of NMR-based metabolomics allows for multidimensional analysis of biomarkers that facilitates unambiguous assignment and quantification of metabolites in complex biofluids.

## 1. Introduction

**Figure 1:**
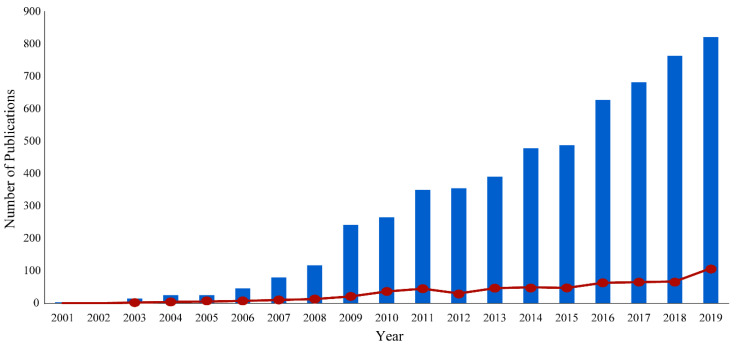
Summary of NMR metabolomic publications (*blue*) and quantitative NMR metabolomic publications (*red*) in PubMed.

Nuclear magnetic resonance (NMR) spectroscopy is an analytical technique routinely used for biomedical metabolomics. NMR has emerged as a valuable application because of its non-invasive, non-destructive, highly reproducible, and quantitative capabilities [1]. NMR can produce qualitative measurements for both known and unknown compounds with little sample preparation [2]. This simple sample preparation allows for minimal alterations to biomedical samples prior to analysis, avoiding bias and biologically irrelevant perturbations [2]. Conversely, other analytical techniques require moderate to extensive sample preparation, chemical derivatization, and time-consuming calibrations to characterize metabolite concentrations. Furthermore, since NMR is a non-destructive technique, a single sample is routinely used to acquire multiple one-dimensional and multidimensional experiments for structural elucidation and metabolite identification. In this regard, NMR may avoid potential sources of inconsistencies or contradictions that may arise from the intrinsic biological variance in replicate samples. Despite these strengths, NMR does encounter several limitations in the analysis of complex biofluids that include lower relative sensitivity and a limited dynamic range. Nevertheless, NMR methodologies continue to evolve and to improve and, accordingly, the application of NMR for the identification and quantification of metabolites has increased dramatically over the past 20 years (Figure 1).

**Figure 2:**
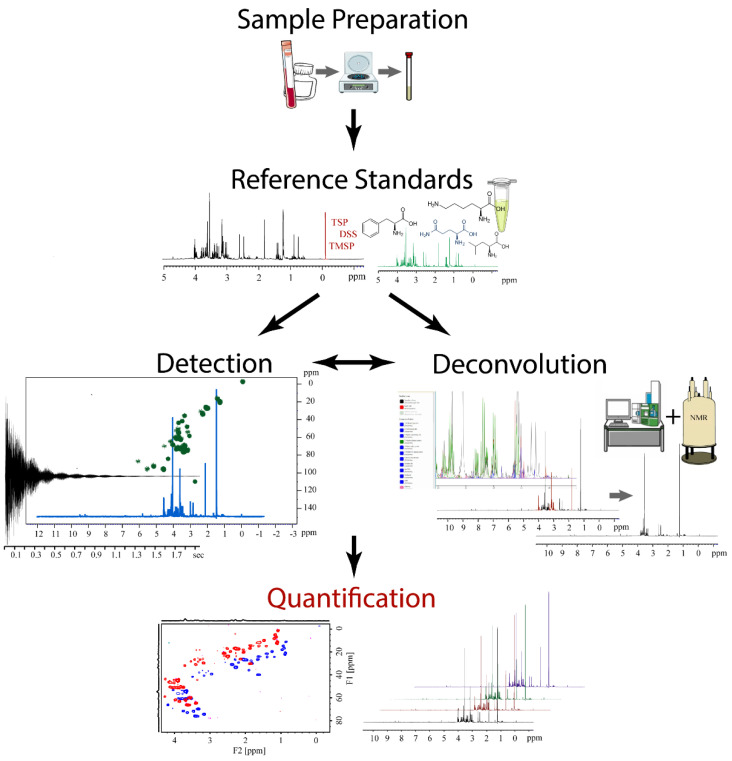
Overview of NMR-based metabolite quantification in biomedical metabolomics. *Step 1*: Sample preparation through deproteinization and/or centrifugation of biofluids. *Step 2:* Selection of reference standard(s) for determination of unknown metabolite concentration. *Step 3*: Detection of analyte signal through NMR spectroscopy. *Step 4*: Metabolite deconvolution by which data is filtered for significant biomarkers of interest. Deconvolution can be achieved through computational methods after data collection and/or experimental design prior to data collection. *Step 5*: Metabolites are quantified by comparison of known metabolite concentration to unknown analytes. The figure was generated using free medical images from Servier Medical Art (https:/smart.servier.com/) under the Creative Commons License Attribution 3.0 Unported (CC BY 3.0).

NMR spectroscopy is inherently quantitative, where signal intensity is directly proportional to the number or count of each individual nuclei (i.e., ^1^H, ^13^C, ^15^N, etc.) present in a sample [3]. This feature makes NMR a natural companion to metabolomics since an overarching goal of the field is to determine the concentration of each detectable metabolite in a biological sample. Quantitative NMR (qNMR) is a specialized subdiscipline within the NMR field that focuses on the highly accurate and reproducible measurements of molecular concentrations [4,5]. qNMR is commonly employed for the analysis of compounds within complex mixtures. Specifically, qNMR has been extensively used for decades within the pharmaceutical industry to quantify active drugs from a variety of complex matrices (e.g., body fluids, drug formulation, natural product extracts, etc.) [6,7,8]. Thus, qNMR is also a useful method for the determination of metabolite concentrations and, accordingly, has seen expanded utilization in metabolomics [9]. A schematic overview of the application of qNMR to metabolomics is shown in Figure 2. The qNMR protocol consists of five steps corresponding to: (1) a simplified sample preparation, (2) selection of reference standards, (3) analyte detection, (4) metabolite deconvolution, and (5) metabolite quantification. The qNMR protocol is highly flexible and allows for the inclusion of multidimensional NMR experiments and the integration of other analytical techniques as appropriate.

There are two basic methods of metabolite quantification by qNMR: relative quantification and absolute quantification [3]. Relative quantification involves the measurement of molar concentrations relative to control samples. This quantification method is commonly used in biomedical metabolomics as a tool to distinguish disease state models from healthy control groups, among other types of comparisons. In NMR-based metabolomics, a relative quantification is commonly achieved through multivariate and univariate statistical analyses of binned NMR spectral data [10]. The resulting statistical models are then utilized to distinguish between the groups and to identify metabolites that vary between the healthy and disease groups. For relative quantification, especially multivariate model analysis, metabolite changes are not independently assessed, but are correlated with other metabolites. Conversely, absolute quantification is the direct measurement of individual compound concentrations independent of all other compounds in the biological sample. Absolute quantification is commonly facilitated by the use of internal standards and metabolite deconvolution [3]. Metabolite deconvolution is achieved in one-dimensional (1D) NMR metabolomics either computationally (i.e., peak fitting) (Section 2.1.6) [11] or experimentally by separating metabolites with analytical methods such as chromatography (Section 2.1.5) [12] or NMR pulse sequences to select metabolites of interest (Section 2.1.3). Deconvolution can also be achieved by multidimensional NMR approaches, in which metabolites are separated along a second dimension by means of through-bond (i.e., HSQC) or through-space (i.e., NOE) correlations (Section 3.1).

Advancements in high-field magnets, cryoprobe technology, and pulse sequence design have continued to push the limits of metabolite quantification below traditional µM concentrations [13]. More importantly, qNMR has been extensively utilized in pharmaceutical studies, where the chemical purity of organic compounds can be estimated with an accuracy and precision of ±1% and an uncertainty less than 0.1% [7]. Furthermore, the implementation of new internal standards and software-assisted metabolite deconvolution techniques has enabled an accurate and broader coverage of metabolites within complex biomedical samples [1,14]. The inclusion of multidimensional NMR, alternative NMR nuclei (e.g., ^13^C, ^15^N, and ^31^P), and solid state NMR has further expanded the utility of qNMR to metabolomics studies [15,16,17]. Additionally, qNMR has found utility in combined analytical techniques, such as HPLC–NMR and NMR–MS [18,19,20,21]. Notably, a broader identification and quantification of the metabolome is achievable by combining multiple analytical techniques that are beyond the capabilities of each individual method. Overall, recent advancements in qNMR technology have significantly improved metabolite quantification, while beneficially impacting metabolomics. In this review, the current status and applications of qNMR, the recent advancements in multidimensional qNMR, and outstanding qNMR challenges and limitations will be discussed.

## 2. Metabolite Quantification in 1D NMR Metabolomics

One-dimensional NMR metabolomics has commonly been divided into two distinct yet complimentary camps: untargeted metabolomics or fingerprinting, and targeted or qNMR. Untargeted metabolomics comprises a simple methodology in which the sample is minimally altered or changed prior to analysis. The chemical composition of an entire biological sample is then characterized or ‘fingerprinted’ in a single experiment. Untargeted metabolomics is thus useful for categorizing biomedical samples, especially disease state identification. Numerous studies have evaluated disease states from global metabolomic changes by comparing disease state models to healthy controls [1,22,23,24]. A predominant feature of the success of high-throughput untargeted metabolomics is the relative quantification of metabolites. Untargeted metabolomics can be readily transformed into qNMR by the addition of internal standards with known concentrations. Simply, the concentration of the internal standard can then be used to quantify the metabolites. Special attention must be paid to the choice of the internal standard(s) used for a specific analysis or sample type.

For 1D qNMR, a number of issues complicate the identification and quantification of metabolites in complex biological solutions. Specifically, peak or spectral overlap, a lack of a universally reliable internal standard, sample size, and the limited dynamic range of NMR may make it difficult to quantify a specific metabolite. Peak or spectral overlap is a serious issue that challenges the quantification of NMR spectra. This problem is particularly pronounced for 1D ^1^H-NMR spectra due to the limited chemical shift range of metabolites (~10 ppm). A ^1^H-NMR spectrum provides a single snapshot of all the detectable compounds (>1 to 5 μM) present in the solution. While this results in a unique fingerprint for every sample, it also means that metabolites with similar chemical structure will appear close together in the spectrum, leading to spectral overlap [25,26]. This situation is further exacerbated by the complexity of the biological samples. The more metabolites that are present in the sample, the more likely spectral overlap will occur. Furthermore, large variations in metabolite concentrations (i.e., μM to mM) will also contribute to the difficulty in detecting a specific metabolite. Simply, an NMR peak from a high concentration metabolite (mM) is likely to mask or obscure a peak for a low concentration metabolite (μM).

Peak overlap due to similar chemical structures is particularly difficult when metabolite quantification of a metabolic pathway is desired. For example, the glycolysis pathway consists of modifications and additions to the core glucose molecule. While each step of glycolysis yields a unique product, there are several repeating chemical scaffolds that result in a significant chemical shift overlap in the 3.2 to 4.5 ppm region of a 1D ^1^H-NMR spectrum. It would be difficult, if not impossible, to quantify a particular metabolite in the glycolysis pathway without deconvolution techniques. Deconvolution of the NMR spectra is a means of removing or isolating metabolites of interest from other peaks that may interfere with the analysis. The deconvolution of qNMR spectra has been accomplished by the application of: 1D NMR pulse sequences (Section 2.1.3), chromatography (Section 2.1.5), computational analysis after data collection (Section 2.1.6), alternative nuclei (Section 2.2.1), two-dimensional (2D) NMR (Section 3), or by chemical additives prior to or during data collection.

One approach to simplify an NMR spectrum would be to completely remove the carbohydrate background by removing all of the carbohydrates from the sample. This was accomplished by selectively oxidizing carbohydrates with sodium periodate followed by removal of the product with hydrazide beads [27]. Other metabolites were not affected by the oxidative removal of the carbohydrates and the resulting NMR spectra were greatly simplified. A similar “Add to Subtract” approach removes the glucose signal by adding a concentrated drop of glucose to a sample post initial data collection, with subsequent collection of a second spectrum. A glucose-free spectrum is created by subtracting the two spectra, which reveals the hidden metabolites lost behind the glucose signal [28]. Another approach of removing metabolites from an NMR spectrum has been accomplished by using nanoparticles to bind specific metabolites [29] or with NMR shift reagents (e.g., gadolinium, Gd^3+^) [30,31] that broaden NMR signals due to T_2_ relaxation. Enzymatic activity has also been utilized to remove signal interference (e.g., removal of urea from urine with urease) [32,33]. An alternative approach is to chemically modify select chemical classes to introduce a distinct ^15^N or ^13^C chemical shift to circumvent the spectral overlap problem. For example, carboxyl and carbonyl groups may be selectively tagged with ^15^N-cholamine [34] or *N*-(2-^15^*N*-aminooxyethyl)-*N*,*N*-dimethyl-1-dodecylammonium [35]. Of course, in all cases, the sample has been significantly manipulated, it is no longer possible to quantify the removed metabolites, and the approach is limited to the chemical removal or modification of a few select chemical classes or compounds. The effect may not be uniform or reproducible across samples or metabolites, and the impact on other metabolites may be difficult to ascertain. Accordingly, the chemical removal or modification of metabolites is not commonly employed by high-throughput NMR-based metabolomics.

### 2.1. Techniques in 1D Metabolite Quantification by NMR

#### 2.1.1. Sample Storage and Preparation for Metabolomics Analysis

The preservation of the metabolomic signature from biofluid samples (i.e., serum, blood, urine, cerebral spinal fluid, and saliva) is an utmost priority. The object of any metabolomics study is to ensure that the analysis accurately represents the intact metabolome of the collected biofluid and avoids any unintended bias. Ideally, the biofluid samples should be analyzed by NMR shortly after collection. If this is not possible or practical, the biofluids are typically stored at −80 °C until analyzed [36]. Multiple freeze–thaw steps should be avoided [37,38]. The preferred pre-analytical procedure for metabolomics analysis involves minimal sample interaction. The simplest sample preparation protocol requires only centrifugation at 4 °C to remove any debris followed by the addition of a deuterated lock solvent (e.g., D_2_O) for NMR data collection [39]. Commonly, a deuterated buffer is used to achieve a constant physiological pH across the entire sample set to minimize chemical shift variation between samples [40]. Another simple technique for biofluid preparation requires treatment with methanol or methanol:chloroform to precipitate biomolecules and prevent further enzymatic activity [41,42]. Ultrafiltration has also been utilized, but may result in significant metabolite loss due to macromolecule binding, and should be used with caution when accurate quantification is desired [42,43]. Tissue samples are also commonly analyzed by solution state NMR, but must be homogenized prior to metabolite extraction [44]. Prior to sample storage, the effective concentration of biofluid samples may be improved by drying the sample and then, reconstituting in a phosphate buffer to the desired sample volume [44]. This is particularly useful when a limited sample is available for NMR analysis, but it does require extra preparation time and may perturb the sample leading to erroneous quantification. In general, the goal of pre-analytical metabolomic extraction is efficiency and consistency. Many protocols require keeping the samples on ice throughout the procedure to avoid sample degradation. Several studies have evaluated the delay time between sample collection, storage, and experimental analysis, as well as the effects of temperature on metabolite degradation. For a comprehensive review of pre-analytical techniques, please see the recent reviews by Bi et al. 2020 [45], Haid et al. 2018 [46], Wang et al. 2018 [47], and Bervoets et al. 2015 [48]. Following pre-analytical preparation, NMR sample preparation requires the addition of phosphate buffer to maintain a physiological pH (pH 7.0–7.4) [40]. Furthermore, maintaining pH levels in sample preparation is beneficial for metabolite identification and quantification against library standards. For a detailed review of high-throughput metabolomics pre-processing protocols for biofluids, please see recent reviews by Vignoli et al. 2019 [40] and Beckonert et al. 2007 [2], for lipid profiling see Barbosa et al. 2108 [49], for tissue extraction see Bhinderwala et al. 2019 [44], and for solid state metabolomics protocols see Tilgner et al. 2019 [50].

#### 2.1.2. Reference Standards for Absolute Quantification

Reference standards are a critical component of qNMR since they are the concentration calibrant for metabolite quantification. There are three common techniques for creating a reference standard in qNMR: (1) an internal reference consisting of a compound directly added to the sample, (2) an external reference consisting of a compound physically separated from the sample, and (3) the Electronic REference To access In vivo Concentrations (ERETIC) method [51]. An internal standard relies on consistent peak heights and peak shapes for all resonances within an NMR spectrum, while an external standard relies on consistent instrument and environmental conditions for all NMR spectra in a dataset [3]. Internal standards have long been used in qNMR techniques due to their high level of accuracy and precision [52,53]. Compounds commonly used as an internal reference for NMR-based biofluid analysis include deuterated trimethylsilylpropanoic acid (TMSP-D_4_), trimethylsilyl propionate (TSP-D_4_), and 4,4-dimethyl-4-silapentane-1-sulfonic acid (DSS). These internal standards are extremely important to metabolite quantification because they provide an identifiable concentration reference that is isolated from areas of significant spectral overlap. Unfortunately, DSS, TMSP-D_4_ and TSP-D_4_ have been observed to bind proteins and other macromolecules found in complex biological samples. Even the presence of a small amount of a protein may cause peak distortion that would lead to inaccurate concentration measurements [2,54,55]. DSS, TMSP-D_4_, and TSP-D_4_ are also susceptible to pH changes since they are weak acids [56]. Formic acid and maleic acid have also been utilized as internal standards and do not bind proteins [2,54,57]. However, formic acid and maleic acid must be used with caution since these are naturally occurring metabolites (i.e., formate metabolism and glycolysis) and may be present in some sample types. In these cases, the inclusion of formic acid or maleic acid may lead to inaccurate metabolite quantification. Octamethylcyclotetrasiloxane (OMS) has also been used as an internal standard for lipid samples [58]. OMS has a boiling point of 448K compared to the more volatile TMS (boiling point 301 K) and has been shown to provide better quantification in chloroform solutions. External standards, on the other hand, eliminate concerns regarding chemical shift changes or peak distortions resulting from molecular interactions or pH differences, while also avoiding peak overlap [3,54]. Simply, separate NMR samples are prepared for the external standard and the biological sample, and NMR spectra are collected under identical experimental parameters and instrumental conditions. Nevertheless, there are also challenges with external standards. For example, external standards drift with time and require multiple calibrations over the course of an experiment to maintain accuracy. Internal and external standards are both routinely used in qNMR. Interestingly, external standards are commonly used by the pharmaceutical industry to determine drug purity levels [59,60], but are less common in metabolomics since internal standards are widely utilized.

ERETIC is an alternative option to using a chemical compound as a reference standard. ERETIC provides an externally calibrated digital signal that creates a reference peak in an NMR spectrum of a complex biofluid sample [51]. Importantly, the frequency of the ERETIC signal can be set to any value within the range of the frequency synthesizer. In this regard, the ERETIC reference signal can avoid peak overlap and can be adjusted, as needed, to appear in any location in the NMR spectrum. Notably, the ERETIC signal is very stable compared to external chemical standards and only requires monthly calibrations [51]. In one study, ERETIC2 (an updated version) and the peak deconvolution software Chenomx were used to generate absolute quantification of metabolites in serum samples [61]. The combined method was defined as software-assisted serum metabolite quantification (SASMeQ). SASMeQ was able to quantify 37 metabolites in serum. ERETIC has also been successfully employed by magnetic resonance imaging [62]. In this study, a 3T MRI system utilized ERETIC to determine the accurate concentration of brain metabolites. ERETIC was shown to be a valid alternative to the commonly used internal water referencing approach in single-voxel magnetic resonance spectroscopy.

In a comparative study of the three quantitation methods, all approaches demonstrated a linear response from 75 to 0.1 mg/mL (1 mg/mL for the internal method) [3]. Conversely, the internal method yielded a better accuracy (−0.1%) compared to the external (−0.3%) and ERETIC (−0.5%) methods. Similarly, the internal method demonstrated a better sample to sample precision (0.3%) compared to the precision of the external (0.7%) and ERETIC (0.6%) methods. Notably, the time-dependent precision (i.e., weekly repeated measurements) of the internal standard method was better than the sample to sample precision. Again, the precision of the internal method (0.1%) was better than the precision for the external (0.3%) or ERETIC (0.3%) methods. Importantly, all three methods were stable for at least a month. Overall, the three quantitation methods yielded acceptable accuracy and precision (<1%) and are equally applicable to qNMR. The external standard and ERETIC methods may be preferred for samples with a large range of concentrations while also avoiding potential protein binding interactions that may occur with internal standards.

#### 2.1.3. NMR Approaches to Filter Macromolecules for Metabolite Quantification

Biofluids contain a large variety of macromolecules (e.g., proteins, DNA, etc.), which may result in significant peak overlap with limited potential for metabolite quantification by qNMR. Pre-analytical procedures such as protein precipitation and ultrafiltration can alleviate overcrowding and simplify the spectral landscape by removing macromolecules. Elimination of macromolecules from biofluids, however, removes the potential for the examination of these other profiles by NMR and multi-omics approaches. Three NMR methods are commonly used to filter macromolecules from the NMR spectrum for metabolomics analysis: Nuclear Overhauser Effect Spectroscopy (NOESY), the Carr–Purcell–Meiboom–Gill (CPMG) pulse sequence, and the diffusion-edited approach [40].

One-dimensional ^1^H NOESY pulse sequence with selective solvent suppression (e.g., H_2_O) is the simplest method of metabolite detection, in which all molecules in the sample are observed, including macromolecules [63]. Accordingly, the 1D ^1^H NOESY experiment requires a pre-analytical filtration step, such as methanol extraction or ultrafiltration, to remove lipids and proteins [42,64]. Alternatively, the CPMG spin-echo pulse sequence enables the detection of small molecular weight molecules within a complex mixture still containing the larger macromolecules. CPMG exploits the large difference in transverse or spin–spin relaxation (T_2_ relaxation) times between small molecules and macromolecules to filter out the NMR signals from proteins and other biomolecules. Notably, the CPMG produces a 1D ^1^H-NMR spectrum similar to a NOESY spectrum, which is obtained following removal of macromolecules by methanol extraction. Nevertheless, the CPMG pulse sequence should be used with caution since it encounters similar problems to ultrafiltration. Any metabolite bound to a protein or other biomolecule will result in a loss of signal and ultimately, an inaccurate quantification. Metabolites, such as tyrosine, histidine, phenylalanine, and tryptophan, that bind to proteins in complex biofluids, have shown significant peak broadening due to a decrease in T_2_ relaxation [65]. The bound metabolite effectively adopts the T_2_ value of the protein and the CPMG effectively filters the protein-bound metabolite. The resulting decrease in peak intensity or peak area for the remaining free-metabolite will result in an underestimation of the metabolite’s true concentration.

**Figure 3:**
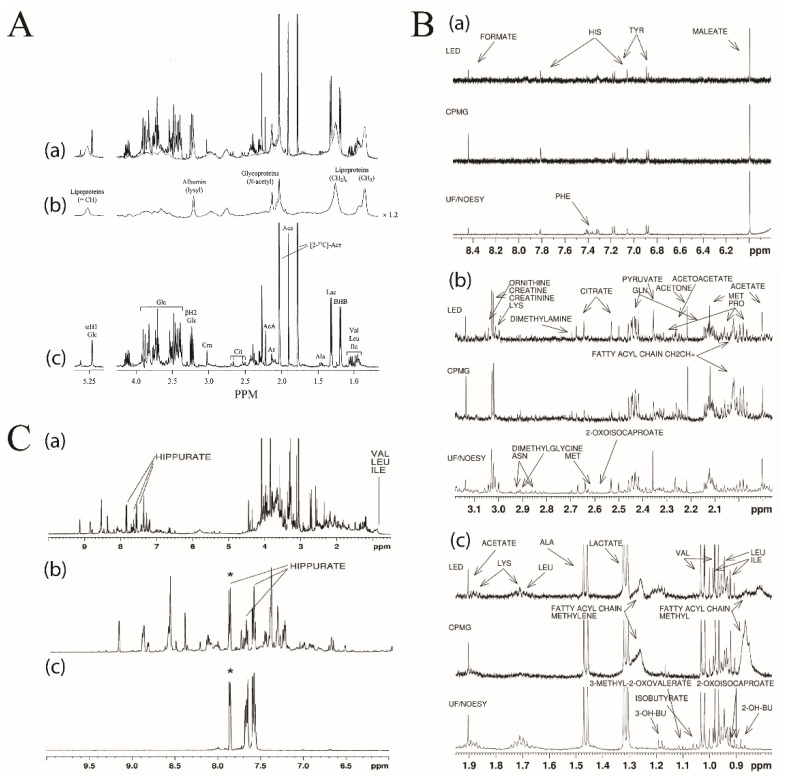
NMR Pulse Sequences for Macromolecular Filtering. (**A**) Diffusion edited approach toward metabolite quantification. (**a**) ^1^H-NMR spectrum of blood plasma acquired with a low sensitivity toward diffusion (b = 4.1 s/mm^2^), overlaid with a spectrum obtained with high diffusion sensitivity (b = 10 000 s/mm^2^, gray). (**b**) ^1^H NMR spectrum of blood plasma acquired with a high sensitivity toward diffusion (b = 10 000 s/mm^2^). The macromolecule spectra in (**a**,**b**) are identical. (**c**) Difference spectrum between (**a**,**b**). Abbreviations are given for acetate (Ace), acetoacetate (AcA), acetone (Ac), alanine (Ala), β-hydroxybutyrate (BHB), citrate (Cit), creatinine (Crn), glucose (Glc), isoleucine (Ile), lactate (Lac), leucine (Leu), and valine (Val). Reprinted with permission from de Graaf, R. A.; Behar, K. L., Quantitative ^1^H-NMR Spectroscopy of Blood Plasma Metabolites. Analytical Chemistry 2003, 75, (9), 2100–2104. Copyright 2003 American Chemical Society. (**B**) Comparison of the different macromolecular signal suppression NMR methods: diffusion edited (LED), T_2_-relaxation edited (CPMG), and the ultrafiltration (UF) of large molecules NOESY NMR spectrum. (**a**) The aromatic region, (**b**) the area from 3.1 to 2 ppm, and (**c**) the area from 0.7 to 1.9 ppm. In each area, the peaks detectable by the LED method are annotated, whereas those only detectable in UF are assigned therein. The most striking differences between methods include the suppression of macromolecular signals and metabolites lysine, ornithine, and phenylalanine that are detectable in LED but not in CPMG spectra. Images reproduced from Bliziotis, N. G.; Engelke, U. F. H.; Aspers, R.; Engel, J.; Deinum, J.; Timmers, H.; Wevers, R. A.; Kluijtmans, L. A. J., A comparison of high-throughput plasma NMR protocols for comparative untargeted metabolomics. *Metabolomics* 2020, *16*. (**C**) Use of semi-selective total correlation spectroscopy (TOCSY) for metabolite quantification. (**a**) Proton NMR spectrum of rat urine acquired using the 1D NOESY Presat sequence to achieve water suppression. (**b**) Low-field expansion of the rat urine proton NMR spectrum. (**c**) Selective TOCSY of rat urine with the selective pulse frequency set on the hippurate 7.88 ppm peak (*) and acquired with SP = 10 ms. Reprinted with permission from Sandusky, P.; Raftery, D., Use of semi-selective TOCSY and the Pearson correlation for the metabonomic analysis of biofluid mixtures: application to urine. *Analytical Chemistry* 2005, *77*, 7717–7723. Copyright 2005 American Chemical Society.

The diffusion-edited pulse sequence also allows for the selective observation of macromolecules in an NMR spectrum [49]. The Longitudinal Eddy-Current Delay (LED) pulse sequence exploits the significant difference in the molecular weight-dependent translational diffusion coefficient between small molecules and macromolecules (Figure 3A) [66,67]. The LED approach produces two distinct spectra. The first spectrum is collected with a low diffusion gradient and, thus, all metabolites and macromolecules are equally represented (Figure 3A, panel a). The second spectrum is acquired with a high diffusion gradient, which results in the removal of metabolites with small diffusion coefficients while leaving macromolecule signals unaffected (Figure 3A, panel b). A diffusion-edited spectrum is produced by subtracting the high diffusion spectrum from the low diffusion spectrum, which results in a spectrum containing only small molecule metabolite NMR signals (Figure 3A, panel c). The diffusion edited approach has been successfully employed for the comprehensive analysis of metabolites or for obtaining lipid profiles for a number of diseases such as lupus nephritis [68] and acute myocardial infraction [69]. A comparative analysis of NOESY, CPMG, and LED showed that LED detected more metabolites then CPMG. The LED results were similar to results obtained with ultrafiltration sample preparation and NOESY pulse sequence (Figure 3B) [64].

In addition to NMR filtration by size, the selective total correlation spectroscopy (TOCSY) experiment obtains a 1D ^1^H-NMR spectrum of a single, preselected metabolite from a complex mixture. The selective TOCSY uses a shaped 180° pulse to excite a single resonance to observe the entire metabolite’s spin system that evolves during the TOCSY spin-lock. The process is repeated for each additional metabolite by the selective excitation of a different NMR peak. As long as the peak chosen for selective excitation does not overlap with other peaks in the NMR spectrum, only a single metabolite will be observed in the resulting TOCSY spectrum. Notably, the other resonances in the metabolite’s spin system may be extensively overlapped in the 1D ^1^H-NMR spectrum, but will be completely resolved in the selective TOCSY spectrum. Thus, selective TOCSY is particularly useful when peak overlap interferes with metabolite detection, as shown in Figure 3C with the detection of hippurate upon a selective pulse at 7.88 ppm [70]. The quantification of low-level metabolites (~ 10 µM) that are at concentrations 1000 times below other major components in a complex mixture is now feasible due to this dramatic spectral simplification [70,71]. It should be noted that the duration of the selective excitation pulse negatively impacts TOCSY peak intensities, which decrease with longer pulse lengths. Furthermore, it is difficult to identify numerous isolated NMR peaks for deconvolution of complex biofluids. Instead, a “semi-selective” TOCSY experiment with shorter excitation pulse lengths will result in a spectrum comprising multiple chemical species, but with better sensitivity. The semi-selective TOCSY is a practical alternative for biofluids [71]. Selective TOCSY experiments have also been used to identify and quantify unknown metabolites in biofluids such as the identification of 4-deoxythreonic acid in human urine [18]. A TOCSY optimized mixture elucidation (TOOMIXED) tool was developed to facilitate the identification of known metabolites in complex mixtures [72]. A series of selective TOCSY experiments are acquired per selective frequency by varying the TOCSY mixing time (*τ*_m_) from 10 to 160 ms. TOCSY peak intensities will modulate as a function of *τ*_m_ and create a unique pattern representative of the metabolite’s molecular structure. In this manner, metabolites may be identified by comparing the matrix of *τ*_m_-dependent normalized peak intensities against a library of known metabolites. A key aspect of the comparison relies on a focused matching based on the selective excitation frequency. The TOOMIXED application does not offer metabolite quantification at this time, but it is an impressive step forward for the structural elucidation of metabolites within complex biofluids.

#### 2.1.4. Sample Size and Limit of Detection

**Figure 4:**
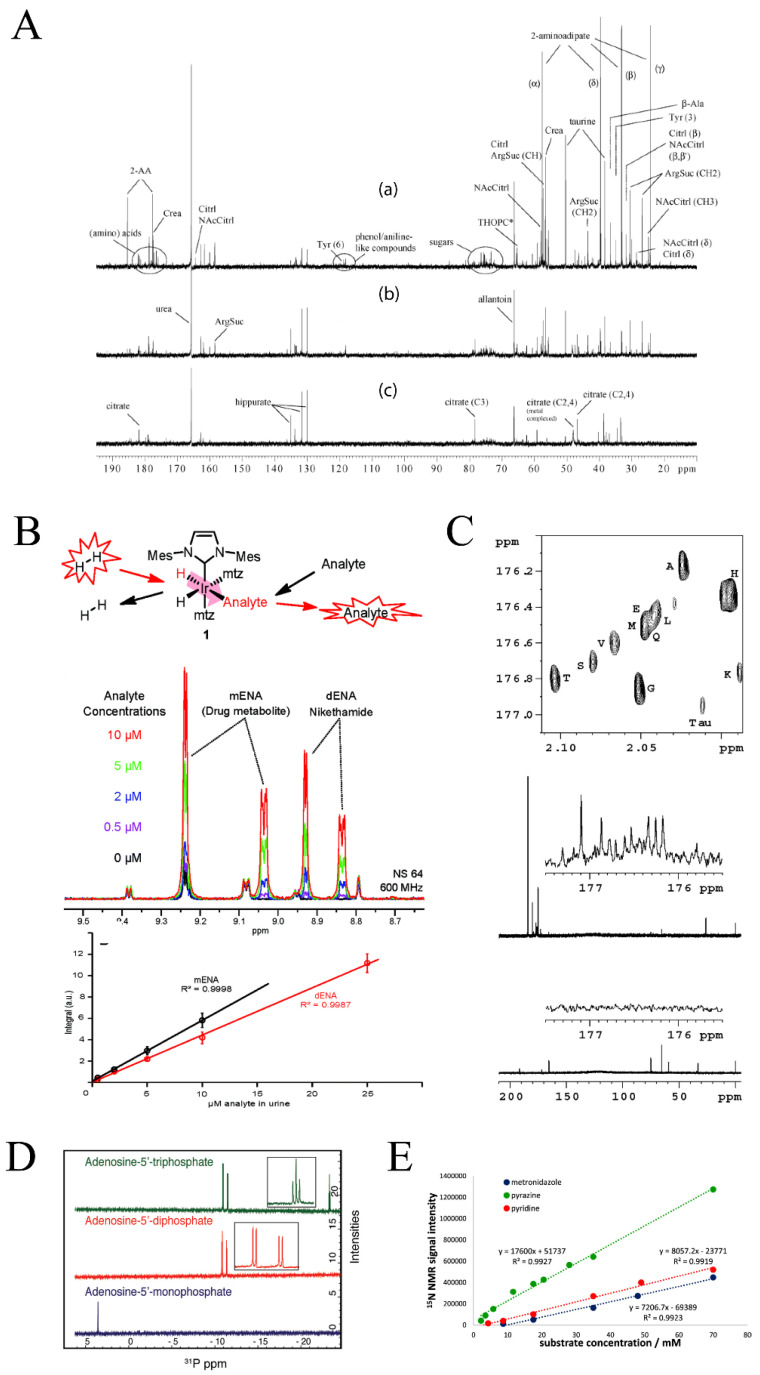
Metabolite Quantification by 1D Solution NMR. (**A**) Typical 500 MHz cryogenic probe ^13^C NMR spectra of rat urine samples taken at 48 h post dose for each dose group: (**a**) control; (**b**) low dose hydrazine, 30 mg/kg; and (**c**) high dose hydrazine, 90 mg/kg. Each spectrum took ~30 min total acquisition time. ArgSuc—argininosuccinate; Citrl—citrulline; NacCitrl—*N*-acetyl citrulline; Citr—citrate; 2-AA—2-aminoadipate; Ala—alanine; Crea—creatine; Crn—creatinine; Tau—taurine; THOPC—1,4,5,6-tetrahydro-6-oxo-3-pyridazine carboxylic acid (*tentatively assigned); Tyr—tyrosine. Reprinted with permission from Keun, H. C.; Beckonert, O.; Griffin, J. L.; Richter, C.; Moskau, D.; Lindon, J. C.; Nicholson, J. K., Cryogenic probe ^13^C-NMR spectroscopy of urine for metabonomic studies. *Analytical Chemisty* 2002, *74*, 4588–4593. Copyright 2002 American Chemical Society. (**B**) Hyperpolarization in qNMR. (*top*) Polarization transfer in signal amplification by reversible exchange (SABRE) schematic. A co-substrate (mtz,1methyl-1,2,3-triazole) is introduced for analyte hyperpolarization in the low-µM regime. (*bottom*) depicts NMR response at different analyte concentrations and calibration curve for drug concentrations dENA and mENA in urine. Error bars represent standard errors of three repeated measurements. Reproduced from Reile, I.; Eshuis, N.; Hermkens, N. K. J.; van Weerdenburg, B. J. A.; Feiters, M. C.; Rutjes, F. P. J. T.; Tessari, M., NMR detection in biofluid extracts at sub-μM concentrations via para-H2 induced hyperpolarization. *Analyst* 2016, *141*, 4001-4005 with permission from The Royal Society of Chemistry. (**C**) ^13^C spectrum of normal human urine (*bottom*), human urine after derivatization with 1,1′ ^13^C_2_ acetic anhydride (*middle*), and 2D HSQC spectrum of derivatized human urine with increased amino acid sensitivity (*top*). Image reproduced from Shanaiah, N.; Desilva, M. A.; Nagana Gowda, G. A.; Raftery, M. A.; Hainline, B. E.; Raftery, D., Class selection of amino acid metabolites in body fluids using chemical derivatization and their enhanced ^13^C-NMR. *Proc. Natl. Acad. Sci. USA* 2007, *104*, 11540–11544. Copyright 2007 National Academy of Sciences. (**D**) Stacked plot spectra of 1D ^31^P spectra for 2 mM solution of AMP (*blue*), ADP (*red*), and ATP (*green*) at pH 4. Image reprinted with permission from Bhinderwala, F.; Evans, P.; Jones, K.; Laws, B. R.; Smith, T. G.; Morton, M.; Powers, R., Phosphorus NMR and Its Application to Metabolomics. *Analytical Chemistry* 2020, *92*, 9536–9545. Copyright 2020 American Chemical Society. (**E**) Raw signal intensity resulting from a series of hyperpolarized naturally abundant ^15^N-NMR spectra of pyridine, metronidazole, and pyrazine as a function of their concentration. The straight lines result from linear regression analysis and the square of the sample correlation coefficient—R^2^ confirms linear behavior. Images reprinted with permission from Fekete, M.; Ahwal, F.; Duckett, S. B., Remarkable Levels of N-15 Polarization Delivered through SABRE into Unlabeled Pyridine, Pyrazine, or Metronidazole Enable Single Scan NMR Quantification at the mM Level. *Journal of Physical Chemistry B* 2020, *124*, 4573–4580. Copyright 2020 American Chemical Society.

Sample volume requirements and limit of detection are also challenges in the application of qNMR to metabolomics. qNMR is limited to identifying the most abundant metabolites in a sample with a limit of detection of approximately ≥3 μM [7]. qNMR also requires a relatively high sample volume of ~500 µL. The intrinsically low sensitivity of qNMR is a result of the narrow energy difference between nuclei spin states. One approach to improve the sensitivity of NMR is to pursue higher magnetic fields; however, this comes with a high price tag and limited accessibility to magnets greater than 18.8T (800 MHz) [73]. Another advancement in NMR sensitivity is the use of cryoprobe technology, where the NMR probe and its electronics are cooled to near liquid helium temperatures (~20K) to reduce electronic thermal noise. As a result, a cryoprobe may lead to a three- to four-fold enhancement in signal to noise. For example, a study examining urinary metabolites associated with drug toxicity used a direct-detect ^13^C cryoprobe, which acquired high quality 1D ^13^C NMR spectra within 30 min that detected numerous metabolites with sufficient signal-to-noise (Figure 4A) [74]. A companion 2D ^1^H-^13^C HSQC spectrum was collected in 4.5 h of naturally abundant (1.1%) ^13^C-urinary metabolites.

In addition to instrumental changes toward sample sensitivity, advancements in sample preparation have led to improved metabolite detection and quantification. NMR sensitivity is directly proportional to sample concentration. However, biofluid samples are often in limited supply, making every µL of available sample extremely valuable. Thus, reducing the required volumes for samples would lead to significant improvements in signal-to-noise by increasing the effective concentration. A popular alternative to improve qNMR sensitivity is to replace traditional 5 mm NMR probes that require relatively large 500 μL sample volumes with either a 3 mm microprobe (150 μL volume) or a 1.7 mm submicroprobe (30 μL volume). A comparison between 5 and 3 mm NMR probes yielded an approximate factor of 2 improvement in signal to noise for the 3 mm probe [75]. A similar comparison between 3 and 1.7 mm probes resulted in another 2.4 factor improvement in signal-to-noise for the 1.7 mm probe [76]. Similar improvements in signal-to-noise can be achieved by using 3 or 1.7 mm NMR tubes in a standard 5 mm NMR probe. Of course, a further enhancement in sensitivity is achieved by combining cryoprobe technology with microprobe technology, such as Bruker’s TCI 1.7 mm MicroCryoProbe^TM^. This approach is particularly advantageous when working with limited samples such as biofluids. Thus, detecting metabolites at submicromolar concentrations is now feasible with micro- or submicro-NMR cryoprobes [77,78].

The most common approach available to an NMR spectroscopist to improve sensitivity is signal averaging, where multiple scans of the same sample are collected in succession and averaged together to produce the final spectrum. This technique, however, increases signal-to-noise at a rate equivalent to the square-root of the number of scans and the acquisition time quickly becomes a practical limitation for high-throughput analysis. This is particularly true for the application of qNMR to metabolomics, where high throughput is a necessity and the number of samples may reach into the thousands. Further exasperating the situation is the requirement to wait 5-times the longest T_1_ value to ensure complete signal recovery for accurate quantitation of the NMR spectrum. Typical ^1^H T_1_ times are in the range of 1 s, but ^13^C T_1_ values can vary significantly, with non-proton bearing carbons having very long T_1_ values on the order of minutes. Thus, qNMR experiments typically strive to minimize acquisition time and the number of scans.

In addition to the limitation of acquisition time, nuclear spin polarization at room temperature with typical magnet fields (14 to 18.8 T) remains relatively low (10^−6^–10^−4^), which explains the low sensitivity of ^1^H and ^13^C-NMR experiments. A polarization transfer from free-electrons has been shown to produce a temporary hyperpolarization state, which achieves a dramatic signal enhancement in the NMR spectrum [79,80]. Parahydrogen-induced polarization (PHIP) of nuclear spins allows for the enhancement of NMR signals based on the conversion of the correlated state of nuclear spins of parahydrogen (i.e., H_2_ or D_2_) via a catalytic reaction, as shown in Figure 4B. This conversion results in a four to five order of magnitude enhancement in the NMR signals [81]. Recently, PHIP was utilized in combination with solid phase extraction to quantify the sub-µM concentrations of drug compounds in urine (Figure 4B) [82]. Hyperpolarization techniques utilizing ^13^C, ^15^N, and ^31^P nuclei are discussed in Section 2.2.1. A targeted qNMR approach combined with hyperpolarization may expand the quantification of dilute compounds in complex mixtures.

#### 2.1.5. Chromatographic Separation Facilitates qNMR

Physical methods of removing spectral overlap are common in qNMR. The combination of high-performance liquid chromatography (HPLC) with NMR spectroscopy has been utilized to successfully isolate and quantify metabolites of interest, with particular benefits to pharmaceutical and drug discovery research [12,83]. Combining NMR and HPLC offers advantages in regions of high spectral interference as well as access to low-concentration metabolites [84]. One of the largest hurdles to overcome when combining HPLC and NMR is solvent interference. Methanol–water and acetonitrile–water are common gradient solvents used to elude compounds from an HPLC column leading to intense peaks in the NMR spectrum. The use of deuterated solvents, such as D_2_O and acetonitrile–D_3_, may reduce the ^1^H solvent signals. Of course, the high volume of deuterated solvents required for HPLC may make this an expensive solution. The saturation of the residual solvent signal is also complicated by the application of gradient elution. Simply, solvent chemical shifts will change in concert with the change in the percentage of each solvent used in the gradient. To address this issue, automatic solvent suppression has been utilized to continually adjust the suppression frequencies as the solvent composition changes [85]. Even if efficient solvent suppression is achieved, ^13^C satellite peaks, which result from the 1.1% natural abundance of ^13^C in H-C bonds and the high solvent concentrations, may also cause an issue in the quantification of low abundant compounds due to spectral overlap [85]. One approach to suppress this interference is to use broadband ^13^C decoupling to collapse the ^13^C satellite peaks.

The low sensitivity of NMR may hinder the application of HPLC to qNMR, but technology advancements continue to decrease the limit of detection in HPLC–NMR techniques [86]. Higher magnetic field strengths (>18.8T), reduced sample volume by employing micro- or submicroprobes [87] with microbore HPLC methods [85], and digital filtering and oversampling during NMR data collection [88] have all contributed to improving detection and quantification in NMR. HPLC–NMR may be used to detect sensitive nuclei such as ^1^H, ^19^F, and ^31^P [85]. The pharmaceutical industry has routinely utilized HPLC–NMR to investigate drug metabolism, and to monitor the effects of drugs or drug candidates on human metabolism, or in animal or in vitro models [85,86,87]. Notably, HPLC–NMR analysis of human urine facilitated the identification and quantification of previously unidentifiable low concentration metabolites, such as 4-deoxythreonic acid [18].

#### 2.1.6. Computational Techniques for Relative and Absolute Metabolite Quantification

The quantification of metabolites is an integral part of NMR metabolomics. One-dimensional ^1^H NMR spectra of metabolomics samples are characterized by a high level of complexity owing to the large number of metabolites present in a biofluid. NMR signals from different metabolites often have similar chemical shifts, which leads to significant spectral overlap. Spectral interference or peak overlap makes the manual quantification of metabolites difficult to achieve [89]. To overcome peak overlap and determine concentration, a reference NMR spectrum needs to be manually adjusted until peak positions and the sum of the peak intensities match the experimental spectrum. This is an exceedingly time-consuming and tedious process that is prone to error. As a result, several automated and semi-automated tools have been developed to assist in the identification and quantification of metabolites in complex mixtures. Many of these software tools have been developed for targeted metabolite comparisons by fitting the experimental spectrum against a compound reference library [90]. Many commercial software packages have cultivated large reference libraries of 1D ^1^H NMR spectra of known metabolites for comparison and analysis against unknown biomedical samples. For example, the Chenomx NMR Suite (Chenomx Inc., Edmonton, Canada) is a commercially available software that offers a large database of common biological and drug metabolite ^1^H 1D NMR data collected over a range of magnetic fields (400 to 800 MHz) and pH values (pH 4 to 9). Furthermore, Chenomx offers supplemental libraries through the open source Human Metabolome Database (HMDB, www.hmdb.ca) [91]. Bruker also offers a BBIOREFCODE 2 database through their AMIX software package that contains a spectral database of 800 compounds acquired at 600 MHz. NMR spectra available in the BBIOREFCODE2 database were acquired at 11 different pH values ranging from pH 3 to 8. The BBIOREFCODE2 database also contains 2D NMR spectra for select compounds at pH 3, 5, and 7. Open source compound libraries including the Biological magnetic Resonance Data Bank (BMRB, www.bmrb.wisc.edu/metabolomics/) [92], the Madison-Qingdao Metabolomics Consortium Database (MMCD, www.mmcd.nmrfam.wisc.edu) [93], and InterSpin (RIKEN, http://dmar.riken.jp/interspin/) [94] contain additional NMR spectra for thousands of compounds collected under various concentration and pH conditions. For example, HMDB houses 1513 compounds with a total of 2862 NMR spectra.

Open source software, Bayesian AuTomated Metabolite Analyzer (BATMAN) [95] and Bayesil [96], were some of the early applications available to combine reference spectra libraries with automated metabolite identification and quantification from 1D ^1^H-NMR spectra. Batman employs a Bayesian approach to assign metabolites in a 1D ^1^H spectrum from a target list and a Markov chain Monte Carlo algorithm to automate relative metabolite quantification [95]. Batman assumes an NMR spectrum of a complex mixture of metabolites is the linear combination of the individual metabolite’s NMR spectra. A target spectrum is matched to the experimental spectrum where peak intensities are scaled, and peak positions are adjusted. On average, Batman is capable of identifying 15 to 20 metabolites, but requires considerable time to complete an analysis (22 min to match 11 metabolites for the 2.3 to 4.1 ppm region) [97]. A detailed review of the Batman workflow can be found at Hao et al. 2014 [98]. Bayesil is comparable to Batman but utilizes probabilistic graphic models to achieve deconvolution of 1D ^1^H-NMR spectra. The Bayesil web server can identify and quantify more than 60 metabolites in serum, plasma, and CSF samples in under 10 min. Bayesil, however, is limited by the sample type and the spectrometer frequency (500 and 600 MHz). In addition, Bayesil has specific requirements for sample preparation and data collection that may be challenging to implement [96]. A comparison of Bayesil and Batman indicated that both methods were useful for quantitative measurements, but required expertise, manual intervention, and extensive computation time [97]. Capabilities available in Batman, namely the ability to add new metabolite reference spectra and the ability to manually adjust a standard metabolite’s spectrum to match the target spectrum, are an advantage over purely automated techniques, such as Bayesil [97,99]. Alternatively, a clear advantage of Bayesil is the minimal computational time and direct readout of concentrations for detected metabolites [96]. Overall, the ready availability of software to automate metabolite quantitation presents a serious risk of over simplifying a complex data analysis and the proliferation of erroneous interpretation [25].

Recent implementations of automated metabolite deconvolution algorithms have focused on high-throughput analysis of NMR metabolomics data [100,101,102]. The automated quantification algorithm (AQuA) was developed to limit the computational time by reducing the amount of NMR spectral data used to quantify metabolites [100]. AQuA simplifies the deconvolution process by using only one unique ^1^H signal, instead of the metabolite’s entire NMR spectrum. Quantitation is then achieved by simple matrix algebra,

(1) $$\bar{\bar{m}}\cdot \vec{x}=\vec{y}$$

where $\bar{\bar{m}}$ is the matrix of the single per-metabolite reference signals, $\vec{x}$ is the vector of peak intensities from the experimental spectrum corresponding to chemical shifts in $\bar{\bar{m}}$, and $\vec{y}$ is the resulting metabolite concentrations. Ration analysis NMR spectroscopy (RANSY), on the other hand, identifies metabolites on the basis of peak height ratios [103]. RANSY exploits the fact that all peak intensities from a given metabolite are proportional to the number of magnetically nonequivalent spins such that peak ratios are fixed and equivalent across spectra. Thus, across a relatively large dataset of NMR spectra (n = 100), the coefficient of variation (CV) of peak ratios from the same metabolite should be theoretically zero compared to the peak ratios between different metabolites given variable concentrations. Like Batman and Bayesil, another method of metabolite deconvolution, ASICS (Automatic Statistical Identification in Complex Spectra), utilizes a library of known metabolite 1D ^1^H-NMR spectra and matches reference peaks to the experimental 1D ^1^H-NMR spectrum [102]. ASICS accomplishes this with a statistical lasso-type estimator. Again, ASICS assumes the NMR spectrum of the complex mixture is a combination of the warped spectra from the library:

(2) $$\sum_{1\leq i\leq p}\alpha_{i}f_{i}\circ \phi_{i}+\varepsilon$$

where *p* is the metabolite library size, *α*_*i*_ is the effective metabolite concentration, *f*_*i*_ is the NMR spectrum, *ϕ*_*i*_ is a warping function to account for chemical shift changes, and ε is an error term. ASICS was shown to outperform open source (e.g., Batman and Bayesil) and commercial software (e.g., Chenomx) in terms of accuracy, as well as an improvement in computational time compared to Batman and Bayesil [102]. However, ASICS, like many automated tools, struggles to accurately assign metabolites in areas of overcrowding and is limited by the number of reference spectral available for comparison [102]. Thus, there is still a need for robust methods to deconvolute 1D ^1^H-NMR spectra and achieve accurate and complete metabolite quantification. Overall, current automated systems lack flexibility to define experimental parameters, while semi-automated systems require expertise to operate and implement effectively.

### 2.2. Applications in 1D qNMR Metabolomics

#### 2.2.1. Alternative Nuclei as a Tool for Quantification

One of the many advantages of NMR spectroscopy is the ability to identify and quantify compounds using multiple nuclei. In principle, it is possible to detect any magnetically active nuclei by qNMR. The most common nuclei used in qNMR are those that have the highest sensitivity and natural abundance (^1^H (99.9885%), ^31^P (100%)) and those that have a large chemical shift dispersion (^13^C (220 ppm), ^19^F (800 ppm)). ^1^H, ^13^C, ^15^N, and ^31^P are also commonly utilized in metabolomics due to their high occurrence in metabolites and biomolecules, where 1D ^1^H NMR is the popular choice. Simply, qNMR experimental parameters are well-established for the acquisition of ^1^H-NMR spectra, and minimal sample preparation or manipulation are required for using ^1^H-NMR in an untargeted metabolomics study. Recent advances in method development and the optimization of experimental parameters have enabled the application of ^13^C, ^15^N and ^31^P-NMR spectra for metabolomics and qNMR [104,105,106,107]. ^13^C-NMR spectroscopy benefits from a broader chemical shift dispersion range but is challenged by a low natural abundance (1.1%). Thus, metabolomics studies utilizing ^13^C NMR commonly incorporate ^13^C-carbons by providing the organism or system a ^13^C-labeled nutrient or metabolite. For example, human urine samples were treated with isotopically labeled 1,1′-^13^C_2_ acetic anhydride to acquire a direct detected 1D ^13^C-NMR spectrum (Figure 4C) [108]. The addition of 1,1′-^13^C_2_ acetic anhydride caused the acetylation of all amines and the incorporation of a ^13^C label into select metabolites. This chemoselective tagging resulted in the identification and quantification of amino acids present in human urine, while eliminating peak overlap common in ^1^H metabolomics studies. Alternatively, NMR experiments of biofluids where ^13^C incorporation is not feasible need to account for the low natural abundance of ^13^C by extensive signal averaging and by using highly concentrated samples, high magnetic fields, cryoprobes, and/or submicroprobes.

^15^N-NMR is also characterized by a broad chemical shift dispersion of approximately 900 ppm. However, the small gyromagnetic ratio, the long relaxation times, and the low natural abundance of ^15^N (0.37%) have limited its application to qNMR and metabolomics. Accordingly, ^15^N-NMR has been primarily used in qNMR as a resonance in a 2D NMR experiment [106]. Conversely, ^31^P-NMR is 100% abundant, with a high gyromagnetic ratio and a moderate chemical shift range of approximately 30 ppm. In fact, ^31^P-NMR saw extensive early usage in metabolomics analysis [109], but has been underutilized of late, potentially because of unfounded concerns regarding chemical shift anisotropy (CSA) effects [110]. While phosphorous-containing metabolites are not as numerous as other nuclei, ^31^P-NMR metabolomics is important to energy metabolism and cell signaling [107]. Energy-related metabolites, such as ATP, ADP, and AMP, can be characterized by ^31^P-NMR (Figure 4D). For example, ^1^H and ^31^P-NMR was used to characterize brain metabolic changes in a rat model of chronic liver disease that resulted in the induction of hepatic encephalopathy [111]. Perturbations in energy metabolism was observed at the end stage of the disease. Specifically, ^31^P-NMR showed decreases in both ATP and ADP 8 weeks post bile duct ligation. In essence, qNMR is not limited to ^1^H-NMR and the choice of nuclei should be based on the specific needs of a given metabolomics investigation.

Hyperpolarization methods have also been applied to other nuclei besides ^1^H [112]. Similar to PHIP, the parahydrogen-based NMR hyperpolarization method, SABRE-Relay (signal amplification by reversible exchange-relay), has demonstrated quantification of low-concentration metabolites with low-abundant nuclei through polarization transfer from parahydrogen [113,114,115]. This technique has been shown to enhance the signal of natural abundant isotopes such as ^13^C (~1.1%) and ^15^N (~0.37%) by a factor of 250 or 39,200, respectively. In this regard, SABRE-Relay allows for collecting a naturally abundant 1D ^13^C or ^15^N spectrum of a millimolar sample with a single scan (Figure 4E). SABRE has also been shown to enhance ^31^P (100%) signals by a factor of 8 [116]. The parahydrogen hyperpolarization technique demonstrated the ability of NMR to exceed inherent limitations of sensitivity and measure metabolites using low abundant nuclei. It should be noted that the detection of naturally abundant nuclei (^13^C and ^15^N) with SABRE-Relay was targeted to nucleobases, peptides, or proteins in high concentration samples. Millimolar concentrations were required for ^15^N detection. Further optimization is required to make SABRE-Relay or other hyperpolarization methods amenable for high-throughput metabolomics analysis of biomedical samples. Instead, isotope labeling and natural abundance quantification are viable alternatives for targeted and untargeted metabolomics investigations using traditional 1D NMR experiments.

#### 2.2.2. Quantification by Solid State NMR Spectroscopy

**Figure 5:**
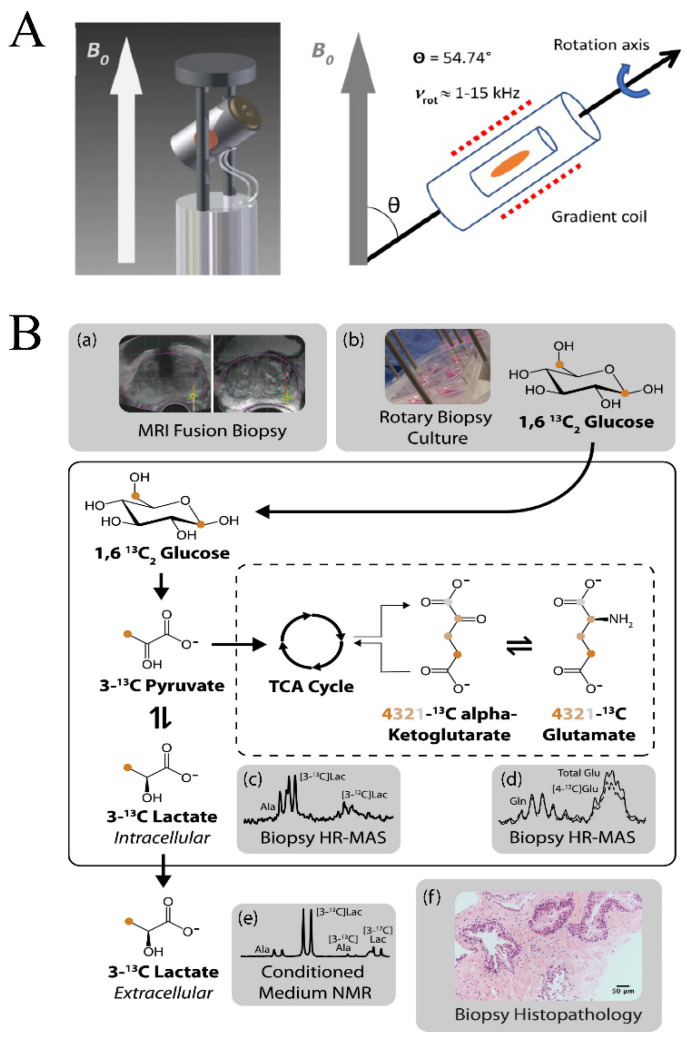
Solid State NMR in qNMR. (**A**) HR-MAS NMR probe head representation with orientation of the stator at an angle (*θ*) of 54.7° between axis of rotation and B_0_. The rotation speed (v_rot_) of the sample reaches up to 15 kHz. A gradient coil is arranged around the rotor. Image reproduced from Gogiashvili, M.; Nowacki, J.; Hergenroder, R.; Hengstler, J. G.; Lambert, J.; Edlund, K., HR-MAS NMR Based Quantitative Metabolomics in Breast Cancer. *Metabolites* 2019, *9*. (**B**) Study protocol for lactate production and efflux in prostate biopsies by NMR quantification. (**a**) MRI fusion biopsy images and (**b**) rotary tissue culture with [1,6-^13^C_2_]glucose supplement. Depiction of tissue lactate (**c**) and glutamate (**d**) concentration and fractional enrichment quantification using HR-MAS of cultured biopsy samples. (**e**) Lactate efflux in media was quantified using solution NMR. (**f**) depicts biopsy histopathology obtained after culture. Images reproduced from Brown, J. B.; Sriram, R.; VanCriekinge, M.; Delos Santos, R.; Sun, J.; Delos Santos, J.; Tabatabai, Z. L.; Shinohara, K.; Nguyen, H.; Peehl, D. M.; Kurhanewicz, J., NMR quantification of lactate production and efflux and glutamate fractional enrichment in living human prostate biopsies cultured with 1,6-C-13(2) glucose. *Magnetic Resonance in Medicine* 2019, *82*, 566–576. Copyright 2019 International Society for Magnetic Resonance in Medicine.

Metabolite quantification by NMR is not limited to the solution state. Recent advances in magic angle spinning magnetic resonance spectroscopy (MAS MRS) have improved metabolite quantification in the solid state. In the solution state, dipolar coupling and the effect of CSA, the orientation dependency of the chemical shift, are negligible due to high molecular mobility and the random tumbling and reorientation of molecules in solution. The only effects measured in solution are the isotopic chemical shift and the through bond coupling that give rise to the appearance of a traditional 1D ^1^H-NMR spectrum. Conversely, in the solid state, molecular mobility is greatly restricted, and individual molecule orientation to the external magnetic field (B_o_) is relatively fixed. Thus, in the solid state MAS MRS analysis, such as a tissue sample, dipolar coupling and CSA do not average to zero, which results in poorly resolved spectra with significant peak broadening [15]. The magnitude of the dipolar constant (*D*) is defined as:

(3) $$D=d\left(3\cos^{2}\theta -1\right);\;d=\left(\frac{\mu_{0}}{4\pi }\right)\frac{\gamma_{I}\gamma_{S}\hbar }{r_{IS}^{3}}$$

where (*θ*) is the angle between B_o_ and the internuclear distance (*r_IS_*) between spins *I* and *S*, *γ* is the gyromagnetic ratio for spins *I* and *S*, *ħ* = *h*/2*π* is Planck’s constant, and *µ*_0_ is the permeability of a vacuum. Spectral broadening is also caused by local magnetic field gradients caused by the tissue sample [117]. In MAS MRS, the solid sample is rapidly rotated at the magic angle (*θ*) of 54.7° (Figure 5A), where dipolar coupling and CSA are averaged to zero, in order to obtain a high-resolution solid state NMR spectrum [118].

Similar to solution state NMR experiments, quantification by MAS MRS requires reference standards. Fortunately, popular internal references (e.g., TSP and DSS) utilized in solution qNMR are also amenable to solid state qNMR [16,119]. However, the same limitations with internal references are encountered in the solid state. TSP and DSS are still prone to bind macromolecules, which includes proteins and lipids that are prevalent in tissue samples. A further loss of the internal standard may occur during MAS MRS sample preparation and rotor assembly, which may lead to an overestimation of metabolite concentrations [119]. Glioblastoma multiforme tumor samples were used to compare the relative performance of an internal standard (e.g., DSS) with the ERETIC method for a MAS MRS qNMR study [119]. DSS-derived metabolite concentrations were consistently higher than the ERETIC measurements. Furthermore, metabolite concentrations had a larger standard deviation when measured by referencing DSS. For example, lactate concentrations were measured as 28.0 ± 9.1 and 50.8 ± 18.9 mmol/kg using the ERETIC method and the DDS internal standard, respectively. Overall, DSS resulted in an overestimation of concentrations by an average of 170%. Similarly, in a study of prostate surgical tissue samples analyzed by MAS MRS and solution state NMR, TSP levels were found to be significantly reduced (71.3% reduction in TSP) when measured by MAS MRS [120]. This discrepancy in TSP levels would thus result in the overestimation of metabolites by MAS MRS. Water resonance has also been utilized as an internal standard in MAS MRS experiments [121]; however, water content is likely to vary between tissues with heavy fat content, such as breast tissues from cancer patients, and thus, can be an inconsistent reference for MAS MRS samples. External standards have also been utilized in MAS MRS as a method of metabolite quantification [122]. As in solution state NMR, ERETIC has demonstrated advantages in MAS MRS applications over traditional internal and external reference standards [16,119,123]. ERECTIC has been successfully used to quantify lactate in living human prostate biopsies cultured with [1,6-^13^C_2_]glucose (Figure 5B) [123]. Glycolysis and TCA metabolites (e.g., lactate and glutamate) were quantified in tissue samples relative to the calibrated amplitude of the ERETIC signal and then further standardized to the wet tissue weight of the sample. Extracellular lactate accumulation was also quantified by TSP with solution state NMR.

One potential issue with MAS MRS quantification is the dependence on tissue weight for metabolite quantification. An underlying assumption of this method is a consistent metabolite level throughout the tissue. Some tissues, such as liver biopsies, have demonstrated a uniformity in metabolite composition throughout the tissue sample, while others, such as tumors, are particularly susceptible to a larger variance in metabolite concentration [124]. For example, a study of intratumoral heterogeneity in breast cancer tissues demonstrated a non-uniform metabolome [124]. Multiple samples were taken from the same tumor which showed significant differences in metabolite quantification across the replicate samples. Specifically, the standard deviation in metabolite concentrations from the breast cancer tissue varied from 0.48 to 0.74 μmol/g. The metabolite concentration variance was significantly higher compared to the standard deviation range of 0.12 to 0.20 μmol/g observed for murine liver tissue samples. It should be noted that while metabolite concentrations varied significantly between biological replicates, this did not limit the ability to predict breast cancer sample identity through Random Forest analysis [124]. A similar concern regarding the spatial variability of metabolites exists with the solution state NMR analysis of tissues. The solvent extraction of a homogenized tissue sample will only provide an overall average of metabolite concentrations. Any spatial variance due to the intrinsic heterogeneity of the tissue structure will be lost. Special attention must be taken to properly quantify metabolites from heterogeneous tissues. Overall, a significant number of ^1^H MAS MRS studies have been conducted to identify and quantify metabolites relevant to various cancers. For a detailed description of the application of ^1^H MAS MRS to metabolite quantification, please see the reviews by Moestue et al. 2011 [125] and Gogiashvili et al. 2019 [126].

#### 2.2.3. Multiplatform Approach to 1D Metabolite Quantification

**Figure 6:**
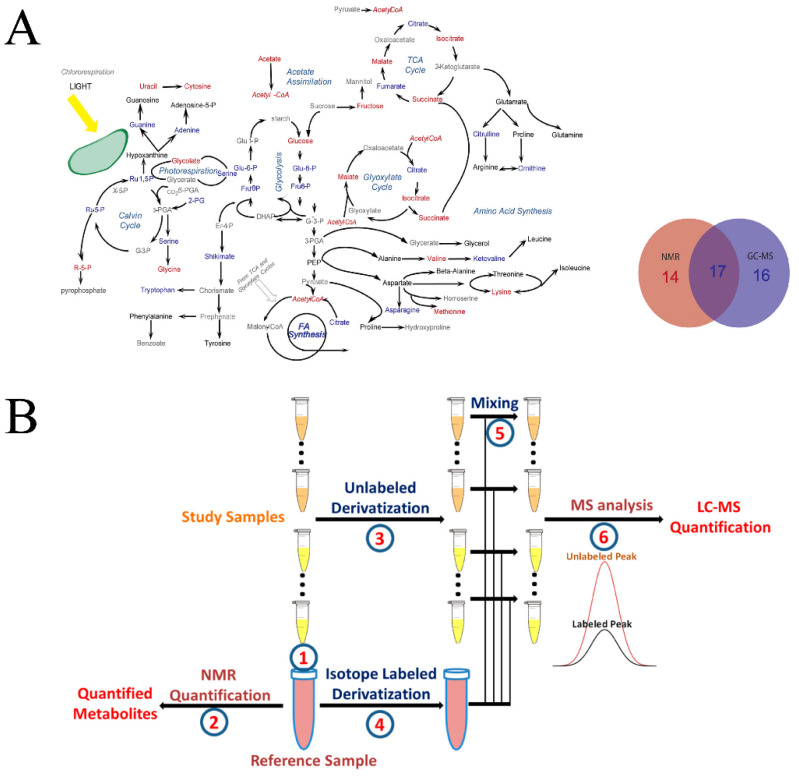
Metabolite Quantification in Combined Metabolomics. (**A**) shows the metabolite pathway summery of *Chlamydomonas reinhardti* metabolome from combined NMR (*red*) and GC–MS (*blue*) techniques. Metabolites identified by both methods are colored black, and metabolites that are not identified are colored gray. The embedded Venn diagram identifies total number of metabolites within these metabolomic pathways. Reprinted with permission from Bhinderwala, F.; Wase, N.; DiRusso, C.; Powers, R., Combining Mass Spectrometry and NMR Improves Metabolite Detection and Annotation. *Journal of Proteome Research* 2018, *17*, 4017–4022. Copyright 2018 American Chemical Society. (**B**) The overall analytical strategy to combine NMR and LC–MS for the absolute quantification of amino acids in serum samples. *Step 1:* Prepare a reference sample with similar matrix to study samples. *Step 2:* Divide the reference sample into two portions. The first portion will be examined to determine the metabolite concentrations using qNMR. The other portion will be used in Step 4. *Step 3*: Derivatize each individual sample under investigation with an unlabeled tag. *Step 4:* Derivatize the second portion of the reference sample with an isotope-labeled reagent (same reagent used in Step 3). *Step 5:* After derivatization, mix each individual sample with the reference sample in a 1:1 (v:v) ratio. *Step 6*: The mixture is then subjected to MS analysis. Given the determined concentrations of metabolites in the reference sample from Step 2, the metabolite concentrations in each study sample can be easily calculated on the basis of the ratio between the labeled and unlabeled MS peaks. Reprinted with permission from Fei, Q.; Wang, D. F.; Jasbi, P.; Zhang, P.; Gowda, G. A. N.; Raftery, D.; Gu, H. W., Combining NMR and MS with Chemical Derivatization for Absolute Quantification with Reduced Matrix Effects. *Analytical Chemistry* 2019, *91*, 4055–4062. Copyright 2019 American Chemical Society.

In general, a multi-omics approach combines genomics, proteomics, metabolomics, and/or lipidomics in order to achieve a thorough characterization of a system [127,128]. Similarly, a multiplatform approach intends to combine multiple analytical techniques to extend cover of the metabolome [129]. Recent advances in multiplatform technologies have demonstrated the combined strength of liquid chromatography/gas chromatography–mass spectrometry (LC/GC–MS) and NMR in the identification and quantification of metabolites. Simply, a multiplatform approach takes advantage of the ease of NMR quantification and the sensitivity of LC/GC–MS. For example, 2D ^1^H-^13^C HSQC NMR was combined with GC–MS to characterize the metabolome of *Chlamydomonas reinhardtii* following treatment with two lipid accumulation modulators (WD30030 and WD10784) [130,131]. In this case, approximately one-third of the metabolites of interest were uniquely identified by NMR, one-third by GC–MS, and one-third by both techniques. Importantly, the metabolites identified by both NMR and GC–MS exhibited a similar response to compound treatment. NMR and MS were shown to be complementary techniques that together achieved a broader coverage of the metabolome and the complete characterization of the metabolic response to a compound treatment that was not possible by either method alone (Figure 6A) [130]. qNMR has also been shown to guide the absolute quantification of metabolites from blood samples using MS [132]. Simply, qNMR was used to quantify 30 metabolites from an arbitrarily identified reference sample from the sample set. The corresponding multiple-reaction monitoring (MRM) peak integral from the mass spectrum of the same reference sample was then used to define the metabolite’s concentration in each mass spectrum in the dataset:

(4) $$C_{(MS)mn}=MRM_{mn}\frac{C_{(NMR)mr}}{MRM_{mr}}$$

where *C*_(*MS*)*mn*_ is the absolute concentration of metabolite *m* in sample *n* based on the MS spectrum, *MRM_mn_* is the MS peak area in the reference sample *r* and *MRM_mn_* is the MS peak area in sample *n* for metabolite *m*, and *C*_(*NMR*)*mr*_ is the absolute concentration of metabolite *m* as determined from the NMR spectrum of reference sample *r*. Unfortunately, the qNMR–MS method is still negatively impacted by matrix effects, which may explain a poor correlation between MS- and NMR-derived absolute concentrations for a subset of metabolites. To address this issue, the qNMR–MS method was refined to include derivatization with and without an isotope-labeled reagent (Figure 6B) [133]. As before, qNMR was used to define the absolute quantitation of metabolites in a reference sample and MS was used to determine the corresponding reference MS peak integral. The reference sample was then derivatized with an isotope labeled reagent, while all other samples in the dataset were derivatized with the same unlabeled reagent. An aliquot of the derivatized reference sample was added to each sample in the test set prior to collecting a mass spectrum. A response factor (*R*) was measured for each metabolite based on a ratio of the peak areas for the labeled and unlabeled MS peaks, which was then used to calculate the absolute concentration of a metabolite in an LC–MS/MS spectrum.

(5) $$C_{Si}=\frac{A_{Si}}{A_{Ri-labeled}}\times R\times C_{R};\;R=\frac{A_{R-labeled}}{A_{R-unlabeled}}$$

where *A_R–labeled_* and *A_R–unlabeled_* are the MS peak areas after derivatization with a labeled and unlabeled reagent, respectively; *C_si_* and *C_R_* are the metabolite’s concentrations in the test sample and reference sample, respectively; *A_si_* and *A_Ri-labed_* are the metabolite’s peak areas in the unlabeled test sample and the labeled reference sample, respectively. A qNMR-guided LC–MS/MS study was able to determine the absolute quantification of four immunosuppressive drugs in human whole blood [134]. The four drugs, cyclosporine A, tacrolimus, sirolimus, and everolimus, were detected over a concentration range of 0.25 to 50 ng/mL with an overall uncertainty of ≤9.0%. Importantly, the four drugs were detected and quantified in under 10 min, while eliminating calibration curves typically required by MS quantification. Furthermore, avoiding the need for a calibration curve is particularly beneficial and may eliminate a practical barrier since numerous metabolites are not commercially available or may be cost-prohibitive to obtain. Several recent studies have utilized a combined NMR and MS platform to identify and quantify biomarkers for neurodegeneration [20], bipolar disorder [135], autism spectrum disorder [136], and prostate cancer [137], among others. The combination of NMR and MS improves the limits of detection, the ease and speed of quantification, and ultimately, expands the coverage of the metabolome beyond the capabilities of either technique alone.

## 3. Metabolite Quantification in 2D NMR Metabolomics

**Figure 7:**
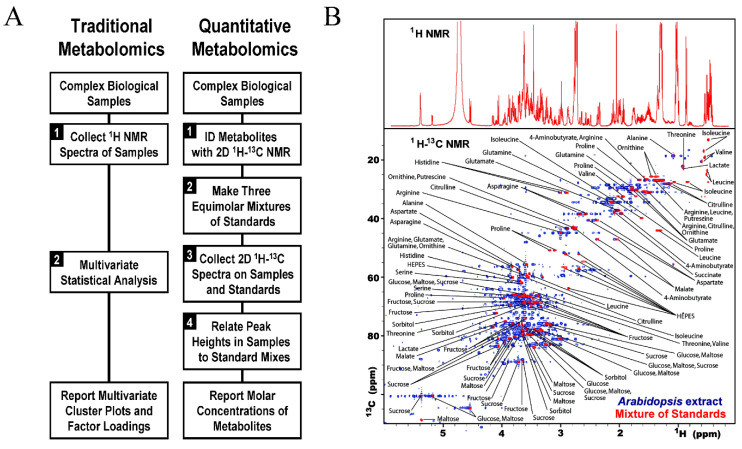
Metabolite Quantification in 2D NMR. (**A**) shows the experimental design used in traditional metabolomics (*left*) and the four-step 2D quantitative metabolomics protocol fast metabolite quantification (FMQ) by NMR. (**B**) depicts (*top*) 1D ^1^H-NMR spectrum of equimolar mixture of small-molecule standards and (*bottom*) 2D ^1^H-^13^C HSQC NMR spectra of the same standard mixture (*red*) overlaid on a spectrum of *Arabidopsis thaliana* (*blue*). Reprinted with permission from Lewis, I. A.; Schommer, S. C.; Hodis, B.; Robb, K. A.; Tonelli, M.; Westler, W. M.; Sussman, M. R.; Markley, J. L., Method for Determining Molar Concentrations of Metabolites in Complex Solutions from Two-Dimensional ^1^H-^13^C-NMR Spectra. *Analytical Chemistry* 2007, *79*, 9385–9390. Copyright 2007 American Chemical Society.

In addition to 1D NMR, multidimensional NMR offers a robust toolbox of qualitative and quantitative methods to detect and quantify metabolites using homonuclear and heteronuclear chemical shifts, spin–spin couplings (J-coupling), dipolar couplings, and through space dipolar–dipolar interactions (NOEs) [8,138]. As outlined in Figure 7A [139], quantitative metabolomics of a complex mixture involves: (1) metabolite identification by 2D NMR analysis, (2) acquisition of reference standards, (3) collection of reference 2D NMR spectra, and (4) quantification of metabolites according to known reference concentrations. As shown in Figure 7B, a major advantage of 2D NMR experiments is the ability to disperse peaks into two-dimensional space and to significantly increase spectral resolution. Essentially, a 2D NMR experiment may be viewed as a simple deconvolution of a 1D NMR spectrum. Furthermore, 2D pulse sequences that exploit J-coupling, dipolar coupling, and/or NOEs to correlate multiple chemical shifts to the same compound provide a means to unambiguously assign known metabolites from complex biofluids. As a non-destructive technique, NMR enables the acquisition of multiple experiments for the qualitative and quantitative characteristics of biological samples. Thus, a combination of multiple 2D NMR experiments have been commonly employed to identify the structure of unknown or novel metabolites [140].

One limitation of 2D NMR experiments is the significant increase in experimental time going from 1D NMR experiments (minutes) to 2D NMR experiments (hours to days), which may be a practical limitation for qNMR studies involving large sample sizes (n > 100). Advances in non-uniform sampling (NUS) are commonly utilized to increase sensitivity, while keeping a constant acquisition time [141,142]. NUS is commonly employed for 2D NMR experiments with conservative 50% data sparsity for metabolomics experiments [143]. Furthermore, a sine-weighted Poisson-gap sampling scheme with the Iterative Soft Thresholding Compressed Sensing method (CS-IST) reconstruction algorithm yielded the best qNMR performance. Specifically, an average CV of 8.03 was obtained for six metabolites over a concentration range from 15.6 to 500 μM [143]. The accuracy and precision of the qNMR experiments were observed to be acceptable according to FDA guidelines. Overall, the application of NUS to metabolomics samples is challenged by the complexity of the samples (i.e., upwards of a 100 or more metabolites), and the diversity of relaxation parameters (i.e., T_1_ and T_2_) across the metabolites. Accordingly, it may be difficult to identify a single set of NUS experimental parameters to accurately quantify all metabolites in a sample. Thus, a conservative approach to employing NUS is recommended. Furthermore, advances in experimental design, NMR pulse sequences, and instrumentation have also shortened the acquisition time for 2D NMR experiments while also improving the limits of metabolite quantification.

### 3.1. Experimental Parameters and Pulse Sequences for Metabolite Quantification by 2D NMR

A variety of 2D NMR experiments have been applied to metabolomics. Common heteronuclear experiments include the heteronuclear (^13^C/^15^N) single quantum coherence (HSQC) experiment that correlates a ^1^H chemical shift with its directly bonded ^15^N-nitrogen or ^13^C-carbon chemical shift (^1^H-^13^C, Figure 7B, or ^1^H-^15^N) [18,139], and the heteronuclear multiple bond correlation (HMBC) experiment that correlates a ^1^H chemical shift with ^13^C chemical shifts that are two and three bonds away [144]. HSQC and HMBC experiments are particularly useful in metabolomics because the signal of the lower sensitive heteronuclei (i.e., ^13^C and ^15^N) is enhanced through nuclear spin polarization from the more sensitive active nuclei (^1^H) through J-coupling. In this regard, chemical shifts of low, naturally abundant ^13^C and ^15^N nuclei are easily and quickly obtained, and directly correlated to ^1^H chemical shifts. In addition, the large chemical shift range of the heteronuclei (>200 ppm) help disperse the overlapped ^1^H chemical shifts. The HMBC experiment is essentially a companion experiment to the HSQC experiment and highlights long range couplings to reveal detailed structural information about the metabolites. Altogether, the combination of an HSQC and HMBC experiment has the potential of providing the complete spin system (i.e., all ^1^H, ^13^C, and ^15^N chemical shifts) for the unambiguous identification of metabolites.

Many metabolomic studies also utilize 2D ^1^H-^13^C HSQC as a tool for metabolite quantification. Typically, HSQC experiments are used to determine only relative metabolite changes because quantification is not as straight forward as 1D NMR experiments. While HSQC peak heights or area are still proportional to metabolite concentrations, they are also modulated by other factors such as differences in coupling constants, relaxation times, and experimental parameters. One potential solution is the fast metabolite quantification by NMR (FMQ) approach. FMQ used a combination of standard reference calibration curves with a non-uniform sampling scheme to decrease sample collection time of 2D NMR experiments [139]. Critically, the reference samples and 2D ^1^H-^13^C HSQC spectra were prepared and collected under essentially identical conditions. The HSQC spectra were collected with a range of metabolite concentrations (2 to 10 mM) and the HSQC peak intensities were plotted as a function of concentration and then, normalized to an internal standard to produce a calibration curve. Approximately 40 metabolites from extracts of *Arabidopsis thaliana*, *Saccharomyces cerevisiae*, and *Medicago sativa* were quantified with an accuracy error of 2.7%.

HSQC_0_ is an alternative approach that addresses the HSQC quantitation problem while avoiding the need for standard calibration curves. HSQC_0_ collects a series of HSQC spectra with an increasing repetition time of the basic HSQC block [145,146]. Each HSQC peak volume is then plotted as a function of the number of HSQC blocks (usually 1 to 3), and then, a best-fit line is extrapolated to time-zero to determine the “true” peak volume. In a similar manner to 1D qNMR, an absolute concentration is obtained by comparing the time-zero peak volumes to an internal standard. Alternatively, a 2D modified ERETIC avoids the need to include an internal reference for calibrating an HSQC experiment [147]. It should be noted that the HSQC_0_ pulse sequence requires a collection time of 8 h for each of the three HSQC spectra (blocks). A total acquisition time of 24 h may limit the utility of HSQC_0_ for high-throughput qNMR. The HSQC_0_ acquisition time was reduced to 3 h per spectrum with the use of a relaxation enhancing agent (e.g., Fe(III)EDTA)) for the analysis of bovine liver extracts [145].

The Q-HSQC [148] and other variants (QQ-HSQC, Q-CAHSQC, Q-OCCAHSQC, etc.) [149,150,151] have addressed the HSQC quantitation issue by replacing a single average value for the one bond ^1^H-^13^C coupling constant with an effective range of coupling constants. This is accomplished by summing signals from a collection of experiments with different values (in a 3:1 ratio of 2.94:5.92 ms) for the INEPT delay (i.e., Δ= 1/2J). This results in a flat, uniform transfer efficiency instead of a peak at 150 Hz (Δ = 3.33 ms), which is the typical average coupling constant and setting used for a 2D ^1^H-^13^C HSQC experiment. As a result, the Q-HSQC acquisition time is four time longer than a standard HSQC experiment [148]. The total reported acquisition time ranged from 12 to 30 h for a model mixture of lignins. The subsequent Q-CAHSQC and Q-OCCAHSQC experiments required a total experimental time of 16 h and 5 min or 15 h and 57 min to measure strychnine, respectively [150,151]. The Quick, Quantitative HSQC (QQ-HSQC) pulse sequence was developed to solve this long acquisition time. The QQ-HSQC pulse sequence utilizes a slice-selective adiabatic sweep to pulse three-fourths of the active region with the shorter delay (∆ = 2.94 ms), while the other fourth was exposed to the longer delay (Δ = 5.92 ms). This sweep pulse preserved the 3:1 ratio necessary for quantification while reducing the acquisition time to 45 min for a sample of strychnine [149]. It should be noted that the Q-HSQC experiments also lead to an overall decrease in transfer efficiency (~30% lost) and spectral sensitivity. Overall, the Q-HSQC and its sequence variations (QQ-HSQC and Q-CAHSQC) deliver a standard deviation of 7 to 9% from expected results [149].

The perfect HSQC pulse sequence replaces that standard INEPT block with a perfect echo INEPT module that suppresses phase distortions due to ^1^H-^1^H J-coupling, leading to further improvements in quantitation [152]. Of course, completely suppressing ^1^H-^1^H J-coupling in an HSQC spectrum would lead to further overall improvement in spectral quality, sensitivity, and ease of quantitation. The quantitative perfected and pure shifted HSQC (QUIPU HSQC) combines a number of recent advances to speed up 2D data acquisition (e.g., NUS, spectral aliasing, and variable repetition time) with a pure shift (i.e., homo-decoupled spectrum) to improve the quantitation of the HSQC experiment [153]. QUIPU HSQC achieved a 6 to 9 factor improvement in data acquisition, a higher quality spectrum due to the removal of ^1^H-^1^H J-coupling, an average trueness of 8%, and a repeatability of 6%. The QUIPU HSQC spectrum, however, still required 5 h to acquire, which would be a challenging limitation for large datasets.

The potential of in vivo metabolite quantification by 2D ^1^H-^13^C HSQC was evaluated with ^13^C isotopically enriched *Daphnia magna* (water fleas) [154]. In this study, the 2D modified ERETIC was combined with the perfect HSQC sequence or the Q-OCCAHSQC sequence to quantify the in vivo concentrations of alanine and phenylalanine. While both pulse sequences yielded comparable quantitation on standard samples, with average root-mean-square deviations (RMSDs) of 4.5 to 6.2%, sample heating and TOCSY artifacts were identified problems with Q-OCCAHSQC. It was also observed that the OC decoupling pulse outperformed standard GARP-4 and CHIRP-95 decoupling pulses with improved bandwidth, no apparent artifacts, and better reproducibility in signal quantification. For the water fleas in vivo samples, the perfect HSQC pulse sequence with optimized parameters yielded RMSDs of 6.0% (alanine) and 2.9% (phenylalanine) compared to 1D qNMR data.

In addition to heteronuclear NMR spectroscopy, homonuclear NMR spectroscopy has also been utilized for metabolite identification and quantification. Common 2D homonuclear experiments used in qNMR include the correlation spectroscopy (COSY) experiment that correlates J-coupled (typically three-bonds) ^1^H chemical shifts [155], the total correlation spectroscopy (TOCSY) that correlates all J-coupled ^1^H chemical shifts in a spin system [71,107], and 2D NOESY experiments that correlate all ^1^H chemical shifts that are dipolar-coupled through space (≤6 Å) [156]. Similar to the HSQC and HMBC spectral pair, the COSY and TOCSY experiments provide complementary information with the potential of obtaining the complete ^1^H spin system for the unambiguous identification of metabolites. An advantage of COSY and TOCSY experiments to qNMR is the intrinsically higher sensitivity of ^1^H NMR relative to other nuclei. Unfortunately, the absolute quantification of metabolites from a COSY or TOCSY spectrum encounters similar challenges with accuracy as occurred with an HSQC spectrum. Again, peak intensities are modulated by differences in coupling constants, relaxation times, and experimental parameters. Furthermore, the integration of COSY and TOCSY cross-peaks may be complicated by complex splitting patterns with multiple antiphase (opposite intensities) peaks separated by the coupling constant. Additionally, this cross-peak shape complexity increases the likelihood of peak overlap and partial peak cancellation. Long experimental times, relative to 1D qNMR, are also a concern with COSY and TOCSY experiments.

A “multi-scan single shot” (M3S) strategy demonstrated a quantitative improvement over a traditional COSY experiment, while decreasing experimental time [157]. The MS3 strategy uses ultrafast NMR techniques that obtain a 2D NMR spectrum with a single scan by employing a spatially encoded pulse sequence [158]. M3S improves quantitation by collecting the single scan COSY spectrum with 256 scans, for a total experimental time of 20 min. The M3S COSY spectrum was shown to be more sensitive compared to a traditional COSY experiment due to the suppression of *t_1_* noise. The M3S COSY was compared to the traditional COSY experiment by quantifying 14 metabolites. Quantification was achieved by using the standard addition method with an added concentration range of 1.0 to 3.7 mM. Simply, a standard sample consisting of the 14 metabolites at a known concentration was added to the breast cancer cell extract and the COSY spectrum was then re-acquired. The process was repeated three times, the peak volumes were plotted as a function of the added metabolite concentrations, and then, a standard addition curve was fitted (V = a[m] + b) to the data to solve the metabolite concentrations ([m]). The M3S COSY achieved a precision error of 1 to 4% compared to 5 to 18% for the traditional COSY experiment. The M3S COSY also had better linearity.

In addition to a COSY analysis, ^1^H-^1^H TOCSY and ^1^H ^−13^C HSQC-TOCSY experiments have also been utilized to identify biomarkers in complex mixtures [159,160]. As previously demonstrated with heteronuclear NMR experiments, the primary motivation of homonuclear NMR experiments has been the identification of metabolites. The ^1^H(^13^C)-TOCCATA database is a repository of ^1^H and ^13^C chemical shift information and spin systems for 455 metabolites, which is widely available for the metabolomic assignment of biomarkers [161]. While quantification of metabolites by 2D analysis has been demonstrated, absolute metabolite quantification has seen limited usage compared to relative quantification.

### 3.2. Software for Quantification of 2D NMR Data: Status and Limitations

Software development for the analysis of 2D NMR metabolomics data has predominantly focused on assignment and deconvolution strategies. Open source data repositories, such as HMDB [91] and BMRB [92], have assembled and curated multiple 2D NMR spectral data for over a thousand known metabolites. HMDB reports the accumulation of 1040 heteronuclear NMR spectra. These databases enable manual, semi-automated, and automated assignments, and are essential resources for software such as COLMARm (CCIC, www.spin.ccic.ohio-state.edu). COLMARm makes use of chemical shifts from ^1^H-^13^C HSQC, ^1^H-^1^H TOCSY, and ^1^H-^13^C HSQC-TOCSY spectra to automate metabolite assignments [162]. Other software such as MetaboMiner (MetaboMiner, www.wishart.biology.ualberta.ca/metabominer) [163] and Spin Assign (RIKEN, www.dmar.riken.jp/spinassign/) [94] are also available for semi-automated metabolite assignments. While several software applications have been developed for metabolite identification, there are fewer software available for metabolite quantification. Several studies have utilized statistical models based on external standard calculations to quantify metabolites [139,154,157] or peak integration of successive experiments [153]. In addition, software within the virtual machine NMRbox (National Center for Biomolecular NMR Data Processing and Analysis, https://nmrbox.org/) [164] has been utilized for relative quantification of 2D NMR metabolomics experiments [20,106,107]. It is evident that while the identification of metabolites by 2D NMR has expanded rapidly in recent years, the development of universally applied quantification methods remain elusive.

## 4. Final Thoughts and Conclusions

The high accuracy and reproducibility of metabolite quantitation and the ease and ready application of qNMR methods makes NMR a unique resource for metabolomics. Metabolite quantification by NMR has seen many advances in recent years, and NMR-based metabolomics continues to be adopted by a variety of fields. While techniques such as LC–MS and GC–MS have dominated metabolomics due to a superior sensitivity, NMR has significantly improved metabolite quantification through the development of reference standards, pulse sequences, physical and chemical deconvolution methods, hyperpolarization techniques, physical and chemical deconvolution methods, and the integration of multiple analytical platforms.

ERETIC and common external and internal standards (e.g., TSP and DSS) have all been extensively utilized in metabolomics quantification. Importantly, each method yielded acceptable accuracy and precision for qNMR, but each approach also has unique advantages and limitations that must be considered during experimental design. For example, the versatility of ERETIC has been shown to benefit many NMR applications, but deconvolution software routinely relies on internal standards for quantification. A number of computational methods for metabolite deconvolution and quantification were discussed. While progress has been made, significant advancements are still needed. For example, semi-automated metabolite assignment is now possible, but the refinement of the identification and quantification of individual metabolites still requires manual intervention by an expert. Open source libraries such as HMDB and BMRB have assembled reference spectra for thousands of metabolites to facilitate accurate metabolite assignments, but the databases are far from complete, where only a small fraction of the predicted or known metabolites have an entry, and each metabolite entry may not have a complete set of standard NMR spectra. The spectral data may also be dispersed across multiple databases. Instead, a single NMR metabolomics database that compiles all available reference spectra with ongoing efforts to acquire missing spectral data would be a preferred alternative.

Simplification of the NMR spectrum may also improve metabolite identification and quantification. The most common approach is to combine NMR with HPLC to isolate and quantify specific metabolites of interest. Alternatively, removal of metabolites or chemical classes can be achieved by solvent extraction, chemical modification, enzymatic degradation, or selective binding (e.g., nanoparticles). Of course, the chemical or physical manipulation of a biofluid is likely to perturb the absolute concentration of a given metabolite [165]. Alternatively, a variety of NMR experiments or approaches may achieve comparable outcomes. The CPMG pulse sequence or diffusion-editing are routinely employed to filter macromolecules from metabolites or lipids. However, metabolite binding to the filtered macromolecules may lead to erroneous quantifications [42]. Multidimensional NMR experiments may remove spectral overlap by spreading NMR signals into two or three dimensions, but at the cost of longer experimental times and potentially lower sensitivity. The application of ^13^C, ^15^N, or ^31^P NMR can expand the resolution of 2D NMR experiments or greatly simplify a 1D NMR spectrum by selecting for metabolites that only contain the specified heteronuclei. Similarly, a selective or semi-selective TOCSY experiment may select a single spin system or metabolite from a complex mixture to improve resolution [70,71], but these approaches may lead to lower sensitivity and an increase in the limits of detection for the qNMR experiment. In all cases, the simplification of the NMR spectra will greatly reduce the number of detected metabolites and may be more appropriate for targeted metabolomics. Overall, the proper choice of a spectral simplification technique will depend on the specifics needs of a given qNMR experiment.

qNMR is also challenged by the intrinsically low sensitivity of NMR spectroscopy and by limitations in sample size. A number of recent instrumentation and methodology advancements have resulted in significant improvements in NMR sensitivity. In addition to higher magnetic fields (1.2 GHz) [73], cryoprobes combined with microprobe or submicroprobe technologies have also significantly improved sensitivity while reducing sample requirements to 30 μL. It is now feasible to detect metabolites at submicromolar concentrations [77,78]. Further advancements in hyperpolarization techniques, such as PHIP and SABRE-Relay, show great promise in enhancing NMR sensitivities by multiple orders of magnitude [81,113,114,115]. Similarly, multiplatform approaches that combine LC/GC–MS with NMR may enable metabolite quantification into the sub-nanogram regime [132]. However, requirements for additional specialized and expensive equipment may limit the broad application or adoption of these techniques.

qNMR has also seen advancements in solid state methodology, improved utilization of nuclei such as ^13^C, ^15^N, and ^31^P for multidimensional NMR, and the adoption of multiple analytical platforms for the expanded coverage of the metabolome. The use of ^13^C or ^15^N-NMR routinely relies on incorporating ^13^C-carbons or ^15^N-nitrogens into the biological samples, which is not usually an option for biofluids. Nevertheless, significant advancements in hyperpolarization techniques are enabling the detection of naturally abundant ^13^C and ^15^N nuclei. While 2D NMR metabolomics has not achieved the same level of high-throughput analysis as 1D NMR, advancements in pulse sequences, NUS acquisition, and applications, such as fast metabolite quantification by NMR, have enabled the inclusion of 2D NMR experiments into metabolomics studies without sacrificing throughput or signal sensitivity. Several 2D NMR pulse sequences have been developed to quantify metabolites through ^1^H-^13^C bond correlation, including HSQC_0_ and many variations of the Q-HSQC approach. These techniques offer an expanded toolkit for metabolite quantification by NMR while eliminating the extensive spectral overlap that plagues 1D NMR. Overall, qNMR is a valuable and integral resource for metabolomics and has benefited numerous applications from drug discovery and disease diagnosis to environmental and nutritional studies.
